# Boundaries and Bridges: The Complex Alliance Between Counselling and Learning Theories

**DOI:** 10.1007/s12124-026-10040-1

**Published:** 2026-10-23

**Authors:** T.B. Borbély-Pecze, John McCarthy

**Affiliations:** 1 Institute for Research on Adult Education and Knowledge Management, Faculty of Education and Psychology, Eötvös Loránd University (ELTE), Budapest, Hungary; 2 Director International Centre for Career Development and Public Policy, Nice, France

**Keywords:** Counselling as learning, Socratic method, Transformative learning, Philosophical foundations, Career counselling

## Abstract

Counselling theories have traditionally been conceptualised within psychological, therapeutic, and developmental frameworks while learning theories have largely evolved as a separate field of inquiry. Although both domains are concerned with personal change, adaptation, and the development of new understandings, explicit theoretical integration between counselling and learning remains relatively limited. This article explores whether counselling may be usefully understood through the lens of learning theory and examines the extent to which key learning concepts can illuminate processes commonly associated with counselling. Drawing on major traditions in learning theory, including behaviourist, cognitive, constructivist, social, experiential, transformative, and humanistic perspectives, the paper develops a conceptual framework for analysing counselling as a context in which intensive personal learning frequently occurs. Rather than proposing an equivalence between counselling and learning, the analysis identifies significant areas of conceptual overlap and highlights how counselling may facilitate changes in knowledge, self-understanding, behaviour, meaning making, and identity. Attention is given to the roles of reflection, experience, dialogue, agency, and relational processes in supporting learning-oriented forms of personal development. The article concludes that a learning perspective offers a valuable complementary lens for understanding counselling processes and provides a basis for strengthening interdisciplinary dialogue between counselling theory and learning theory. Implications for theory development, research, and professional practice are discussed, alongside the limitations of the present conceptual analysis.

## Defining Counselling and Scope

In this paper, counselling is understood as a structured helping relationship intended to facilitate client self-understanding, decision making, adaptation, meaning construction, personal development, and action. While the practice of psychotherapy, counselling psychology, and counselling differs in important respects across national, professional, and institutional contexts, they all involve purposeful dialogical interventions aimed at facilitating change.

Career counselling and career guidance are focused upon in this paper because these fields provide clear examples of learning-oriented change processes involving identity development, meaning construction, adaptability, and agency. While the discussion draws extensively on career-counselling literature, the conceptual arguments developed here are intended to apply more broadly across counselling as a family of helping practices. Examples from other counselling traditions are therefore incorporated where relevant. The argument concerns counselling as a broad family of helping practices rather than a single professional model.

## Defining Learning and Scope

Learning is broadly understood in this paper as the process through which individuals modify or reconstruct knowledge, skills, meanings, identities, values, understandings, or patterns of action through experience, reflection, dialogue, and participation in social practices (Illeris, 2018; Mezirow, 1991). Learning is therefore not restricted to formal education or the acquisition of information, but includes transformative, experiential, sociocultural, affective, and identity-related processes of change.

While the discussion draws substantially on adult learning theory, particularly because counselling most often engages adults in processes of self-understanding, decision-making, and life transition, the argument is not confined to a single learning tradition. Rather, the paper incorporates insights from constructivist, transformative, experiential, sociocultural, and dialogical theories of learning insofar as they help explain how change occurs within counselling relationships (Bruner, 1996; Illeris, 2018; Kolb, 1984; Mezirow, 1991; Vygotsky, 1978). This broader conception provides the conceptual basis for examining counselling as a learning-intensive practice. Contemporary educational theorists have further argued that learning should be understood not merely as acquisition but as participation in processes through which individuals come into relationship with others and the world (Biesta, 2022).

Clarifying the Problem: A Taken-for-Granted Theoretical Separation.

Counselling theory and learning theory come from distinct academic and institutional traditions, despite sharing similarities. Although significant conceptual overlaps have been identified among constructivist, career-development, and career-learning literatures, efforts to develop an explicit and comprehensive theoretical framework that systematically integrates these domains remain limited.

Theories of counselling have evolved mainly within the context of counselling psychology, clinical psychology, and psychotherapy, and have been concerned with processes of change, sense-making, and well-being (Corey, 2021; Norcross & Lambert, 2019). Theories of learning, on the other hand, have their antecedents in educational psychology, instructional science, and experimental psychology, and have been mainly concerned with the acquisition and transformation of knowledge, skills, and understanding via experience (Illeris, 2018; Sfard, 1998).

Despite this separation, many counselling theories describe change through concepts such as perspective transformation, meaning reconstruction, identity development, and new ways of understanding experience, notions that closely resemble contemporary learning theories (Mezirow, 1991; Rogers, 1961). Recent developments in career construction theory, life design counselling, and contemporary career-development scholarship increasingly challenge this separation and suggest the value of understanding counselling-related change as a learning process (Maree, 2022; Wang & Li, 2024).

Learning theory emerged from philosophical debates about knowledge, experimental psychology’s search for lawful mechanisms of change, and educational concerns with instruction and efficiency. While early traditions prioritised observable behaviour and cognition, later constructivist and sociocultural approaches re-centred meaning, dialogue, and identity bringing learning theory conceptually closer to counselling (Table 1).

**Table 1:** Disciplinary alignment of early career guidance and learning theory

Early career guidance/Career development	Learning theory
Rooted in industrial psychology and vocational guidance	Rooted in educational, experimental, and developmental psychology
Aligned with trait–factor approaches	Focused on mechanisms of learning and change
Emphasis on ability testing and psychometrics	Emphasis on learning processes and outcomes
Focus on stable individual differences	Focus on development, plasticity, and adaptation
Key question: What kind of person fits which occupation?	Key question: How do people change through learning experience?
Counselling framed as assessment and decision support	Instruction framed as facilitation of learning
Change assumed to occur through counselling	Change assumed to occur through the learning intervention
Typical contexts: employment services and guidance offices	Typical contexts: classrooms, training, adult education

This divergence helps explain why early career counselling theory aligned more readily with psychometric and trait–factor models, while learning theory developed separately within educational contexts concerned with processes of change and development.

## Existing Connections Between Counselling and Learning

Constructivist, narrative, career-construction, and systems-oriented approaches have long conceptualised counselling as a process of learning, meaning construction, identity development, and adaptation (Peavy, 1997, 2000; Guichard, 2005, 2009; Patton & McMahon, 2014; Savickas, 2013). Across these perspectives, counselling is viewed not merely as problem solving or decision support but as a developmental process involving reflection, narrative reconstruction, identity learning, and active meaning making.

These approaches place learning at the centre of counselling practice, although they do not typically engage directly with broader learning theory.

Recent developments in life-design counselling and career-construction theory further reinforce learning-oriented interpretations of counselling by emphasising narrative reconstruction, adaptability, identity development, and self-authorship (Wen et al., 2022; Wang & Li, 2024).

Beyond career-development scholarship, psychotherapy researchers have similarly described therapy as a context for transformative, affective, and meaning-related learning, further supporting the relevance of learning concepts for understanding counselling-related change (Rose et al., 2005; Rossiter, 2022; McCullough & Long, 2024).

## Counselling as a Deliberate and Intensive Learning Process

This article does not seek to argue that counselling involves learning, a proposition already recognised in several counselling traditions. Rather, it seeks to develop and strengthen a more explicit dialogue between counselling theory and learning theory by examining how counselling-related change may be interpreted through educational, transformative, experiential, sociocultural, affective, and dialogical perspectives on learning.

First, it identifies the historical separation between counselling theory and learning theory. Second, it examines counselling as a learning-intensive process. Third, it uses the Socratic-dialogical tradition as a bridge concept linking counselling and learning. Finally, it evaluates the limits of this framework and proposes a broader conception of counselling as relational, experiential, sociocultural, and transformative learning.

In the light of the previous discussion on the separation of disciplines, we propose in counter-argument that counselling may be usefully understood not only as support or facilitation, but also as a context within which intensive personal learning frequently occurs.

In various traditions of counselling, the process of client change is often conceptualised in terms that are remarkably like learning outcomes. Clients are encouraged to develop new conceptual frameworks for understanding their circumstances, substituting habitual or limiting frameworks with more differentiated and adaptive models of understanding (Beck, 2011; Bruner, 1996). In both cognitive and constructivist models of counselling, the process of counselling itself consists of helping clients identify, assess, and modify meaning systems that structure perception and action, a process that is remarkably like the principles of conceptual change in learning theory (Illeris, 2018).

Counselling further enables the building of new selves through the reconstruction of personal narratives. Recent developments within life design and career construction traditions have explicitly framed these narrative processes as central mechanisms of career-related change. Clients are encouraged to actively author their future trajectories through reflective storytelling, identity exploration, and the construction of meaningful life themes (Wen et al., 2022; Wang & Li, 2024). These developments provide contemporary support for understanding counselling as a learning-intensive process rather than solely a problem-solving intervention.

Counselling may also facilitate the development of new behavioural repertoires through experimentation, practice, and reflection, extending learning beyond changes in understanding to changes in action (Corey, 2021; Kolb, 1984).

Taken together, these processes suggest that counselling may be understood as a learning-intensive intervention involving conceptual, narrative, behavioural, affective, and transformative forms of change. Viewing counselling through this lens does not reduce it to learning but provides a useful framework for understanding how change occurs through counselling relationships.

## Counselling as Personal Learning: What Kind of Learning?

If counselling is understood as a learning-intensive process, a central theoretical question follows: what kind of learning occurs within counselling relationships? Although counselling scholarship provides rich accounts of change in terms of identity development, narrative reconstruction, agency, adaptability, and wellbeing, the learning processes underpinning such changes are not always explicitly theorized as forms of learning (Illeris, 2018; Mezirow, 1991; Zalaquett et al., 2013). Clarifying the nature of this learning therefore provides an important foundation for understanding counselling as a learning-intensive practice.

One form of learning evident in counselling and counselling-related change is instrumental learning, involving the development of skills, coping strategies, and behavioural repertoires that support more effective functioning. Behavioural and cognitive-behavioural traditions make these learning processes particularly explicit (Beck, 2011; Corey, 2021).

In addition to instrumental, skill-based outcomes, counselling can be a matter of transformative learning, whereby clients transform their meaning perspectives rather than accumulate new skill sets.According to transformative learning theory, learning becomes transformative when individuals critically reflect on assumptions that previously shaped their understanding of experience and, through dialogue and reflection, develop new interpretations and perspectives (Mezirow, 1991). Transformations, in counselling settings, might occur if clients begin to see long-held beliefs or self-interpretations as partial, distorted, or no longer adequate, resulting in more complex, flexible understandings about self and situation (Illeris, 2018).

Identity learning is another aspect of counsellor-mediated change whereby clients work to modify their conception of themselves and what they might become. Narrative therapies have long conceptualized counsellors as involved in assisting a re-authoring of the self, in which clients can make sense of themselves through a cohesive life story (McAdams, 2001; Savickas, 2013). In this perspective, learning in a counsellor-mediated way is inextricably linked with identity formation, whereby changes in identity are predicated on changes in self-narrative. Counselling can also be understood as facilitating forms of existential and spiritual learning, especially within the humanistic and existential traditions. Such approaches conceptualise counselling as an encounter in which individuals engage with questions concerning meaning, responsibility, freedom, values, and purpose, rather than focusing exclusively on symptoms or behavioural adjustment. (Rogers, 1961; Yalom, 1980). Taken together, these forms of learning suggest that counselling frequently operates as a form of second-order learning in which individuals not only learn new skills or perspectives but also examine how they make meaning, interpret experience, and construct knowledge about themselves and the world (Bateson, 1972; Mezirow, 1991). This distinguishes counselling from many conventional learning contexts by focusing on the transformation of underlying meaning frameworks rather than the acquisition of information alone.

Although much of the learning literature discussed above focuses on reflection, meaning making, and conceptual change, counselling-related learning is not exclusively cognitive. Counselling as affective learning is exemplified in person-centred counselling, gestalt therapy, and existential counselling, which treat the therapeutic or educational process as an experience driven by emotions, values, and relational dynamics rather than purely cognitive functioning. It recognises that emotions play a pivotal role in shaping cognitive processes, memory retention, decision-making, behaviour, and in their transformations through counselling. Individuals frequently learn through emotional experience, embodied awareness, relational encounters, and processes that remain partly outside conscious reflection. The unconditional positive regard of the counsellor in person-centred therapy (Rogers, 1961), aimed at facilitating client change, is of itself an affective posture and value stance as well as an intellectual stance. Psychodynamic, attachment-oriented, and relational traditions have long suggested that change may occur through new emotional experiences, experiences of recognition, and the development of more secure relational patterns rather than solely through explicit insight (Schore, 2012; Stern & Boston Change Process Study Group, 1998). Similarly, feminist, Indigenous, and spiritually informed perspectives often emphasise connection, relationality, and experiences of meaning that extend beyond rational reflection alone. Understanding counselling as learning therefore requires attention not only to cognitive transformation but also to affective, embodied, relational, and sometimes existential or spiritual dimensions of human development.

## The Socratic Method as a Bridge Concept

A useful bridge between counselling theory and learning theory can be found in the Socratic-dialogical tradition. The Socratic method provides a model of learning based on guided inquiry rather than direct instruction. Through questioning, individuals are encouraged to examine assumptions, identify contradictions, and develop their own conclusions (Nelson, 1949; Plato, trans. Grube, 2002). By encouraging individuals to examine assumptions and tolerate uncertainty, Socratic dialogue creates conditions for reflection, learning, and perspective transformation (Bruner, 1996; Nelson, 1949; Plato, trans. Grube, 2002) Fig. 1.

**Fig. 1:**
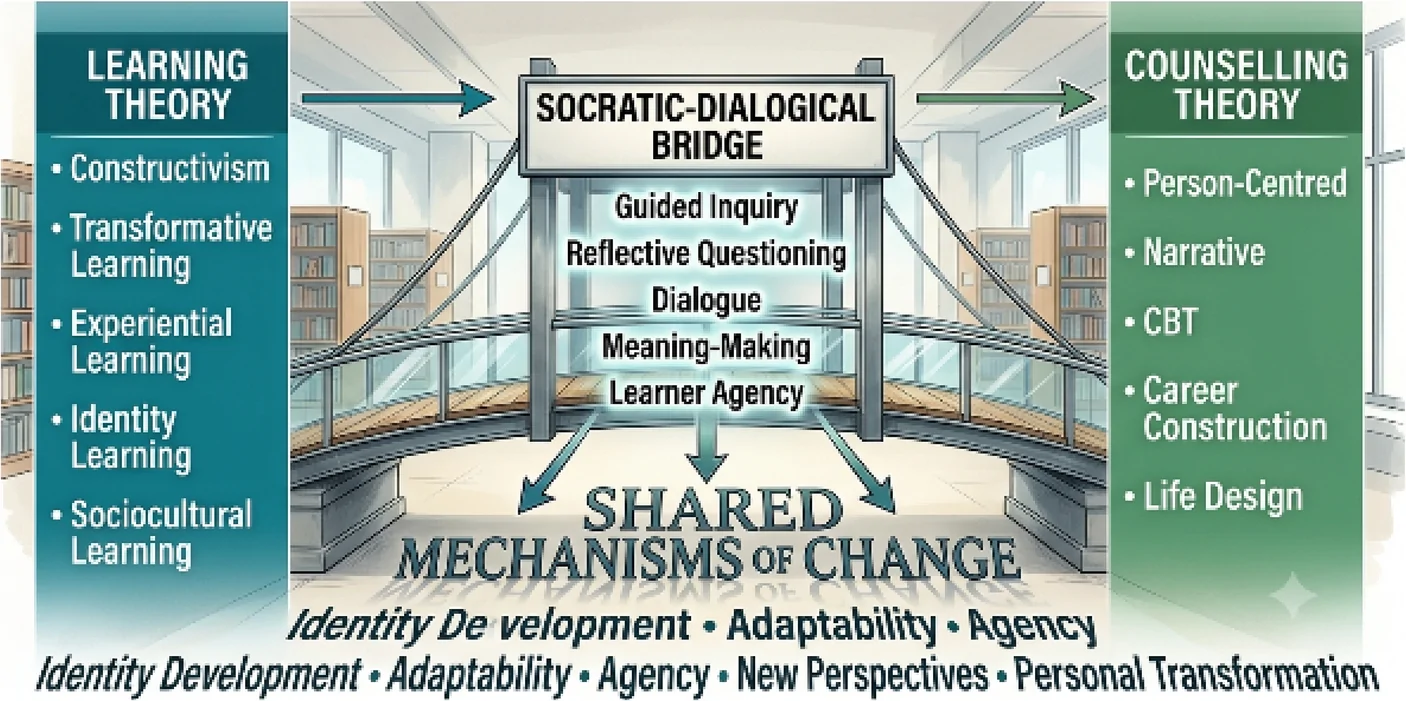
The Socratic – Dialogical bridge between learning theory and counselling theory

The figure illustrates how constructivist, transformative, experiential, identity, and sociocultural theories of learning connect with person-centred narrative, cognitive-behavioural, career-construction, and life-design approaches through shared processes of guided inquiry, reflection, dialogue, meaning making, and learner agency. These processes contribute to changes in identity, understanding, adaptability, and action.

From this perspective, counselling professionals can be observed as operating according to a discernible Socratic logic. In the person-centred, existential, and cognitive traditions, the counsellor often works with the client through questioning rather than through direct instruction or the giving of advice (Corey, 2021).

This Socratic heritage is explicitly recognized in CBT (Cognitive Behavioural Therapy/Counselling) or in Cognitive–Behavioural Career Counselling (CBCC) (Krumboltz, 1996) where there is a specific “Socratic questioning” procedure. In CBT, for example, the counsellor employs specific questions to enable the client to become aware of cognitive distortions and to test their beliefs against the evidence and construct alternative explanations that the client can accept as more adaptive (Beck, 2011). While CBT usually follows a more formal and problem-solving approach than Socratic dialogue, the focus on guided discovery and not persuasion indicates that the goal of developing learner-owned insight is shared.

At the same time, a Socratic approach can be discerned as being implicit in humanistic and person-centred therapies even when a language of technique is rejected. The emphasis on empathy, congruence, and unconditional positive regard does not diminish direction but reconfigures it in a relational form of facilitation that recognizes the autonomy of the client while being supportive of self-examination through reflection (Rogers, 1961). In this way, humanistic therapies embody a Socratic approach in which dialogue serves as a forum where a re-evaluation of understanding can take place without formalizing it as a technique.

The value of the Socratic framework lies in illustrating how structured dialogue can facilitate learning, insight, and change while preserving learner agency. At the same time, important limitations remain, particularly regarding affective, relational, and non-conscious dimensions of counselling.

Contemporary dialogical educational theory provides additional support for viewing counselling through a Socratic lens. Recent work has emphasized dialogue as a process through which participants jointly construct meaning, develop new understandings, and negotiate identity rather than merely exchange information (Biesta, 2022; Wegerif, 2023). Such perspectives reinforce the argument that learning in counselling is fundamentally dialogical while extending classical Socratic ideas into contemporary educational contexts.

### Counsellor as Socratic Teacher: Strengths of the Analogy

On one level, the analogy illustrates how counselling enables learning through discourse rather than transmission. In this regard, the function of the counsellor is akin to the function of the Socratic teacher, who guides the inquiry, but the learner retains ownership of the conclusions.

In both counselling and transformative learning, moments of dissonance may challenge established interpretations and create opportunities for restructuring meaning. Experiences often described as insight, realisation, or breakthrough can therefore be understood as significant episodes of learning (Mezirow, 1991; Bruner, 1996).

Viewed through a Socratic lens, counselling can be understood as a process of guided inquiry in which learning occurs through dialogue, reflection, and learner-owned insight. The framework therefore provides a useful conceptual bridge between counselling theory and learning theory while preserving the agency and autonomy of the individual.

### The Limits of the Socratic Framing

Although the Socratic approach offers valuable insights into counselling-related learning, it also has important limitations.

The first is related to the issue of power asymmetry. Whilst Socratic dialogue can be conceived as more equalized, it must not be forgotten that the questioner maintains considerable control over the context and tempo, and over the nature of valid insight (Nelson, 1949). Likewise, within the context of counselling, it is the counsellor who maintains control over the context and boundaries of the encounter, and who determines when reflection is appropriate and implicitly evaluates the coherence or adequacy of developing insights. This asymmetry, it should be noted, cannot be eliminated within supposedly nondirective models, but can only be ethically managed (Norcross & Lambert, 2019). A strictly Socratic model may well underestimate this power issue within counselling and fail to capture its ethical complexity.

A second limitation relates to affect and embodiment. The Socratic dialogue model emphasizes cognitive exploration and discursive thinking, with emotion and embodiment as background. However, several counselling approaches have in common a recognition that emotional, somatic, or traumatic aspects of experience are key to counselling processes (Damasio, 1994; van der Kolk, 2014). Learning in counselling settings often involves affect regulation, felt safety, or implicit relational experiences rather than discursive thinking. The Socratic model of dialogue does not lend itself to explaining such processes.

Related to this, counselling may also entail nondiscursive processes of change. Studies on procedural learning, repair of attachment, and attunement indicate that substantial counselling change can and does occur with little or no conscious insight or understanding (Schore, 2012; Stern & Boston Change Process Study Group 1998). Clients may develop new behavioural and relational patterns through experiential processes, including emotional co-regulation, modelling, attachment repair, and repeated interpersonal interaction, rather than solely through questioning or reflection. Such processes are difficult to accommodate within a Socratic tradition that emphasizes conscious examination and questioning.

A third consideration is the cultural applicability of the Socratic approach to learning and meaning making. The Socratic approach to learning and meaning-making rests on a certain type of individualism, in which self-expression, belief scrutiny, and individual action are emphasized. However, these values and principles may not be universalizable to other cultures in which knowledge is conceived of in relational, traditional, or communal terms (Markus & Kitayama, 1991; Rogoff, 2003).

Critiques of the Socratic tradition have also emerged from postcolonial scholarship, feminist pedagogy, intercultural education, and the growing literature on epistemic justice. These critiques suggest that the assumptions underlying classical dialogical models of learning may not be universally applicable and may reflect Western understandings of knowledge, agency, and human development (Biesta, 2017; Freire, 1970).

One concern is that the Socratic model privileges forms of learning centred on rational argumentation, self-reflection, and verbal articulation. While these practices have considerable value, they may overlook other ways of knowing that are relational, embodied, narrative, spiritual, or community based. Postcolonial scholars have argued that educational and helping professions have frequently universalized Western epistemological assumptions while minimizing alternative traditions of knowledge and meaning making (Andreotti, 2011; Santos, 2014). From this perspective, learning cannot be reduced to the critical examination of propositions but must also include collective memory, cultural inheritance, affective lived experience, and locally situated forms of knowledge.

Relatedly, feminist educators have questioned whether models of dialogue based primarily on rational critique adequately capture the relational dimensions of learning. Feminist pedagogy emphasizes care, mutuality, emotion, embodiment, and the co-construction of knowledge, arguing that learning frequently occurs through relationships rather than through intellectual challenge alone (Hooks, 1994; Noddings, 2013). Such perspectives resonate strongly with counselling traditions that regard empathy, attunement, and emotional safety as essential conditions for meaningful change. From this perspective, learning in counselling may emerge not only through questioning and cognitive restructuring but also through experiences of recognition, validation, and relational support.

The literature on epistemic justice offers a further challenge to purely Socratic framings. Fricker (2007) argues that individuals can experience epistemic injustice when their knowledge, testimony, or interpretive resources are marginalized because of their social position. Within counselling contexts, this implies that clients may not merely need help examining beliefs but may also require support in articulating forms of knowledge that have historically been discounted or silenced. A counselling process informed by epistemic justice therefore recognizes that learning is not simply a matter of correcting misunderstanding but may involve restoring legitimacy to overlooked experiences and voices.

These critiques are particularly relevant in multicultural counselling contexts. Indigenous scholars and practitioners have emphasized that knowledge is frequently embedded within relationships, communities, traditions, and ecological systems rather than located solely within individual cognition (Kovach, 2009; Wilson, 2008). Learning therefore may occur through storytelling, observation, collective practice, ceremony, and participation in cultural life rather than through critical dialogue alone. Such perspectives challenge assumptions that self-disclosure, analytical reflection, or argumentative exchange represent universal pathways to development and learning.

Taken together, postcolonial, feminist, intercultural, and epistemic critiques do not invalidate the Socratic framework. Rather, they suggest that it should be understood as one culturally situated account of learning among many possible accounts. The value of the Socratic tradition lies in its ability to illuminate important dialogical and reflective aspects of counselling, but its explanatory power is strengthened when situated alongside broader understandings of relational, embodied, communal, and culturally diverse forms of learning.

Lastly, the Socratic model has very little explanatory power for learning without awareness. There is considerable research in psychology and in psychotherapy that indicates learning does occur through nonconscious processes like habituation, emotional conditioning, and observation learning (Bandura, 1977; Schore, 2012). Counselling-related learning may therefore occur even when clients are not fully aware of the processes through which change has taken place. This learning cannot be conceptualized within a model that places a high premium on reflection as the learning mechanism.

Taken cumulatively, these criticisms imply that while the Socratic method gets something right about counselling, namely its dialogical, reflective, and agency-affirming character, it does not get it all right. An awareness of its limitations maintains the utility of the analogy while preventing it from becoming too comprehensive.

## Toward a Broader Concept of Counselling as Learning

Instead of ending the analysis with a critical perspective, a more fruitful approach is to locate the Socratic framework within a broader conception of counselling as a process of learning. While the Socratic-dialogical tradition illuminates important dimensions of counselling-related change, counselling may be more comprehensively understood as a learning environment in which relational, experiential, sociocultural, institutional, and transformative processes intersect.

First, counselling may be understood as a relational learning process in which change emerges through attunement, interaction, and the co-construction of meaning within the counselling relationship (Buber, 1970; Norcross & Lambert, 2019). From this perspective, learning is not situated exclusively within the individual but is produced through relationships with others. Such an understanding enables attachment-oriented, relational, and interpersonal approaches to be interpreted as forms of learning through experience with the other rather than through reflection alone.

Second, counselling shares important characteristics with experiential learning. Learning often occurs through action, experience, experimentation, emotional processing, and subsequent reflection rather than through abstract instruction (Kolb, 1984). Many counselling approaches encourage clients to test new behaviours, engage with difficult emotions, and develop alternative ways of acting in the world. In this sense, counselling frequently involves learning through lived experience rather than the acquisition of formal knowledge.

Third, counselling may be understood through sociocultural and situated perspectives on learning. Sociocultural and cultural-historical traditions emphasize that learning is mediated through language, relationships, cultural tools, and participation in social practices (Lave & Wenger, 1991; Vygotsky, 1978). From this perspective, development occurs through engagement with others, participation in communities, and interaction within wider social systems (Engeström, 2001). Learning therefore emerges not only within individuals but also through participation in social and cultural environments.

These perspectives are particularly relevant to counselling because they position learning as a dialogical, relational, and socially situated process. The counselling relationship can be viewed as a form of guided participation through which clients develop new interpretations, identities, and possibilities for action. Such approaches extend the analysis beyond individual reflection and reinforce the view that counselling-related change is simultaneously cognitive, relational, cultural, and situational (Biesta, 2022; Rogoff, 2003; Wegerif, 2023).

Counselling-related learning is also shaped by the institutional and cultural contexts within which it occurs. Opportunities for learning vary across public employment services (PES), educational guidance settings, rehabilitation programmes, workplace career development initiatives, and therapeutic contexts, where professional mandates, policy frameworks, available resources, and organisational priorities influence the nature and scope of learning opportunities available to clients. While some settings permit extensive reflection and transformative learning, others focus more narrowly on adaptation, decision making, or labour-market transitions.

Similarly, assumptions concerning expertise, authority, self-disclosure, autonomy, and appropriate forms of interaction vary across cultures. Clients and counsellors may hold different expectations regarding advice-giving, collaboration, emotional expression, and the legitimacy of questioning established beliefs. Counselling-related learning therefore cannot be understood solely as an individual psychological process but must also be recognised as socially, institutionally, and culturally mediated (Ivey & Daniels, 2016; Markus & Kitayama, 1991; Rogoff, 2003).

Recent social-justice approaches to career guidance have further emphasized how power, inequality, and social structures shape opportunities for learning and career development. Rather than focusing exclusively on individual reflection and personal agency, these perspectives highlight the broader social and economic conditions within which identities and careers are constructed (Hooley et al., 2017). From this viewpoint, learning in counselling must be understood as both a personal and a social process.

Finally, counselling may be understood as a transformative learning process through which individuals reformulate assumptions about themselves, others, and the world. Contemporary transformative learning scholarship increasingly emphasises that such transformation is embedded in biography, relationships, emotion, embodiment, imagination, and sometimes spiritual experience, extending learning beyond purely cognitive reframing of assumptions (Formenti & West, 2018; Illeris, 2018; Mezirow, 1991).

Taken together, these perspectives suggest that counselling may be productively understood not as a single form of learning but as a complex learning environment in which affective, relational, experiential, sociocultural, institutional, and transformative processes intersect. Such a conception preserves the insights offered by the Socratic tradition while situating them within a broader account of how learning and change occur in counselling relationships.

## Limitations of the Present Analysis

Several limitations of the present analysis should be acknowledged.

First, this article is an exploratory conceptual and theoretical contribution rather than an empirical investigation. The arguments advanced are based on the interpretation and synthesis of literature drawn from counselling theory, learning theory, career-development scholarship, and related philosophical traditions. Consequently, the paper does not provide direct empirical evidence demonstrating that all counselling processes can be understood as forms of learning, nor does it seek to test the propositions developed here empirically.

Second, the article does not employ a systematic review methodology. The literature discussed has been selected because of its relevance to the conceptual argument rather than through formal systematic-review procedures. While an effort has been made to engage with influential and contemporary scholarship across multiple traditions, the analysis should therefore be understood as an integrative theoretical synthesis rather than a comprehensive review of all relevant bodies of literature.

Third, much of the discussion is rooted in Western traditions of counselling, psychology, philosophy, and educational theory. Although the paper incorporates sociocultural, postcolonial, feminist, Indigenous, and epistemic-justice perspectives, these traditions are represented primarily as critical interlocutors rather than as fully developed alternative frameworks. Different cultural understandings of learning, knowledge, personhood, and helping relationships may generate interpretations of counselling that diverge substantially from those explored here.

Fourth, the Socratic-dialogical tradition has been employed as a heuristic and bridge concept rather than as a comprehensive explanatory model. The value of the Socratic perspective lies in its capacity to illuminate reflective, dialogical, and agency-oriented dimensions of counselling. However, important aspects of counselling-related change, including affective, embodied, relational, tacit, and non-conscious forms of learning, cannot be fully explained through a Socratic framework alone.

Finally, counselling takes place within diverse institutional, professional, and social contexts that shape the opportunities available for learning and development. The extent to which counselling functions as a learning-intensive process may vary across settings such as public employment services, educational guidance, rehabilitation, workplace counselling, and therapeutic practice. Future research may therefore benefit from examining how learning processes are influenced by specific institutional arrangements, cultural conditions, and professional practices. The present analysis could be extended by investigating empirically how different forms of learning emerge within counselling relationships and by exploring whether the conceptual framework developed here is applicable across diverse cultural, institutional, and practice contexts.

A further limitation concerns the conceptual breadth of both counselling and learning. Each field encompasses multiple theoretical traditions, professional practices, and cultural interpretations. Consequently, the framework proposed here should be viewed as a heuristic and exploratory model rather than a definitive account of all forms of counselling or learning.

## Conclusion: Counselling as a Learning-Intensive Practice

The principal contribution of this article is the development of an exploratory conceptual framework that brings counselling theory and learning theory into dialogue with each other, identifying areas of conceptual overlap that may support new theoretical, research, and practice-oriented perspectives.

Contemporary developments in life design counselling, career construction theory, social-justice guidance, and dialogical education all point toward increasing convergence between counselling and learning perspectives (Wang & Li, 2024; Wen et al., 2022; Wegerif, 2023). Rather than representing isolated theoretical traditions, these developments suggest an emerging interdisciplinary conversation concerning how individuals learn to construct identity, agency, meaning, and future possibilities through guided reflection and dialogue.

Recent career-guidance scholarship has increasingly emphasized the challenges presented by labour-market uncertainty, non-linear careers, technological change, and social inequality. These conditions create a growing need for counselling processes that support adaptability, resilience, identity learning, and meaning construction rather than simple occupational matching (Blustein, 2019; Wang & Li, 2024). Consequently, the learning dimension of counselling may become increasingly important in contemporary guidance practice.

This paper has argued that counselling can be usefully understood as a learning-intensive process involving the transformation of meaning, identity, affect, behaviour, and self-understanding. Although counselling theory and learning theory have historically developed within different disciplinary and institutional traditions, there is significant conceptual overlap. Many counselling approaches—including constructivist, narrative, humanistic, cognitive, and career-development traditions—describe processes of change that closely resemble those discussed in theories of transformative, experiential, identity, and situated learning.

At the same time, the paper has argued that counselling should not be reduced to education, teaching, or instruction. Rather, learning provides an additional conceptual lens through which counselling-related change can be understood. The value of this perspective lies not in replacing established counselling theories but in offering a framework that helps explain how changes in understanding, meaning, agency, and identity occur through counselling processes.

The Socratic-dialogical tradition was examined as one possible bridge between counselling theory and learning theory. This tradition highlights the importance of inquiry, reflection, dialogue, and learner agency in facilitating change. However, the analysis also demonstrated important limitations of the Socratic framing, particularly with respect to power relations, emotional and embodied processes, cultural diversity, epistemic justice, and forms of learning that occur outside conscious reflection. Consequently, the Socratic model is best understood as a valuable but partial account of counselling-related learning rather than as a universal explanation of all counselling processes.

A broader understanding of counselling as learning therefore requires attention to relational, experiential, sociocultural, and contextual dimensions of change. Counselling occurs within institutional, historical, and cultural settings, and the opportunities for learning available to clients are shaped by these conditions. Whether taking place in career guidance services, educational settings, public employment services, rehabilitation programmes, or therapeutic contexts, counselling involves processes through which individuals reinterpret experience, develop new possibilities for action, and negotiate evolving identities.

Several implications follow from this perspective. First, counsellor education may benefit from greater engagement with contemporary learning theories, particularly those concerned with transformative, sociocultural, and identity-related forms of learning. Second, counselling research may benefit from examining learning processes directly rather than focusing exclusively on behavioural or symptom-based outcomes. Third, stronger theoretical dialogue between counselling scholars and learning theorists may generate new insights into the mechanisms through which change occurs in helping relationships.

Counselling and learning are not identical phenomena. Important learning processes frequently occur within counselling and learning theory offers a useful complementary perspective for understanding how personal change, reflection, meaning-making, and development take place through counselling relationships. Future empirical research is needed to examine the extent to which the conceptual associations proposed here are observable across different counselling contexts, populations, and cultural settings.

## Data Availability

No datasets were generated or analysed during the current study.

## Declarations

### Clinical Trial

The clinical trial number is *not applicable* for the article as it is a theoretical one.
